# Misfolded protein oligomers induce an increase of intracellular Ca^2+^ causing an escalation of reactive oxidative species

**DOI:** 10.1007/s00018-022-04513-w

**Published:** 2022-08-27

**Authors:** Giulia Fani, Chiara Ester La Torre, Roberta Cascella, Cristina Cecchi, Michele Vendruscolo, Fabrizio Chiti

**Affiliations:** 1 Department of Experimental and Clinical Biomedical Sciences, Section of Biochemistry, University of Florence, Viale Morgagni 50, 50134 Florence, Italy; 2 Centre for Misfolding Diseases, Department of Chemistry, University of Cambridge, Cambridge, CB2 1EW UK

**Keywords:** NMDA receptors, AMPA receptors, Membrane destabilization, Calcium homeostasis, Oxidative stress, Protein misfolding, Neurodegenerative diseases

## Abstract

Alzheimer's disease is characterized by the accumulation in the brain of the amyloid β (Aβ) peptide in the form of senile plaques. According to the amyloid hypothesis, the aggregation process of Aβ also generates smaller soluble misfolded oligomers that contribute to disease progression. One of the mechanisms of Aβ oligomer cytotoxicity is the aberrant interaction of these species with the phospholipid bilayer of cell membranes, with a consequent increase in cytosolic Ca^2+^ levels, flowing from the extracellular space, and production of reactive oxygen species (ROS). Here we investigated the relationship between the increase in Ca^2+^ and ROS levels immediately after the exposure to misfolded protein oligomers, asking whether they are simultaneous or instead one precedes the other. Using Aβ_42_-derived diffusible ligands (ADDLs) and type A HypF-N model oligomers (OAs), we followed the kinetics of ROS production and Ca^2+^ influx in human neuroblastoma SH-SY5Y cells and rat primary cortical neurons in a variety of conditions. In all cases we found a faster increase of intracellular Ca^2+^ than ROS levels, and a lag phase in the latter process. A Ca^2+^-deprived cell medium prevented the increase of intracellular Ca^2+^ ions and abolished ROS production. By contrast, treatment with antioxidant agents prevented ROS formation, did not prevent the initial Ca^2+^ flux, but allowed the cells to react to the initial calcium dyshomeostasis, restoring later the normal levels of the ions. These results reveal a mechanism in which the entry of Ca^2+^ causes the production of ROS in cells challenged by aberrant protein oligomers.

**Supplementary Information:**

The online version contains supplementary material available at 10.1007/s00018-022-04513-w.

## Introduction

Alzheimer’s disease (AD), which is the most common neurodegenerative disease and the most common form of dementia, is characterized by the extracellular deposition in the brain of the amyloid β (Aβ) peptide in the form of senile plaques [1] and by the intraneuronal formation of neurofibrillary tangles of the tau protein [2]. The aggregation process of Aβ generates a large variety of protein aggregates, such as oligomers, protofibrils and fibrils, all characterised by high levels of polymorphism [3]. According to the amyloid hypothesis, the small diffusible oligomers of Aβ are neurotoxic and are thought to contribute to AD development and progression [3–5]. Oligomer cytotoxicity appears to result, in its early phases, from the aberrant interactions of such species with a number of molecular targets on neurons, including the lipid bilayer of their cell membranes [1, 3, 5, 6]. This interaction results in the disruption of cell membranes, compromising its ability to maintain cellular homeostasis, and promoting two important early biochemical changes. The first is the uncontrolled increase in cytosolic calcium (Ca^2+^) levels flowing from the extracellular space into the cytosol [7–14], and the second is the accumulation of reactive oxygen species (ROS) [10, 11, 15, 16].

It is known that Aβ oligomers are able to interact and insert into the phospholipid bilayer of the cell membrane causing the passage through it of small molecules and ions, such as free Ca^2+^ ions [6, 7, 11, 17, 18], as well as permitting the activation of ionotropic glutamate receptors functioning as Ca^2+^ channels, including the *N*-methyl-d-aspartate (NMDA) receptors [9, 14, 15, 18–21] and the α-amino-3-hydroxy-5-methyl-4-isoxazolepropionic acid (AMPA) receptors [14, 15, 18, 19, 22]. In particular, the rapid oligomer-induced activation of extrasynaptic NMDA/AMPA receptors is a crucial mechanism in the AD pathogenesis. This process takes place through the insertion of the oligomers in the bilayer, which changes the mechanical properties of the membrane that is transmitted down to the receptors that are therefore activated through their mechanosensitivity, without a direct interaction with the oligomers [14]. Other Ca^2+^ channels that seem to be involved in the Aβ-induced flux of Ca^2+^ ions are the transient receptor potential melastatin 2 (TRPM2) [23], the voltage-dependent Ca^2+^ channels (VDCCs) [24], and the transient receptor potential A1 (TRPA1) [25].

Another relevant early biochemical change resulting from the interaction of Aβ oligomers with cell membranes is oxidative stress, which is associated with the accumulation of ROS in the cytosol [10, 11, 15, 16] and represents an important determinant in AD pathogenesis [26, 27]. The elevation of ROS is caused by the activation of the oxidative metabolism to respond to the intracellular Ca^2+^ increase induced by the oligomers and the consequent increased need for ATP by the Ca^2+^ pumps, that try to restore the normal levels of intracellular Ca^2+^ [10, 28]. It was also observed that Aβ aggregation can induce oxidative stress though intra-mitochondrial mechanisms, with disruption of the electron transport chain that initiate ROS production [29–31], or with the suppression of α-ketoglutarate dehydrogenase [32]. On the other hand, some studies have proposed that the oxidative stress precedes Aβ accumulation and may therefore induce amyloid production [29–31]. Analysis performed in murine AD models with human overexpression of the amyloid precursor protein (APP) showed that lipid peroxidation and oxidative damage occurs before Aβ accumulation [33]. Moreover, using HEK293 human embryonic kidney cells, it was observed that ROS produced in mitochondria drove Aβ production [34]. Eventually, therefore, Aβ aggregation may be both a cause and an effect of oxidative stress.

It is not yet clear, however, if interactions and cause-and-effect relationships between ROS production and Ca^2+^ signalling induced by misfolded protein oligomers can be considered as bidirectional, or whether one of them is causative of the other. Oxidative stress has been considered, at least in part, a consequence of Ca^2+^ entry into cells, because of the increased need to produce ATP by mitochondria to pump out Ca^2+^ ions, which produces ROS itself [10, 28, 35]. On the other hand, ROS can significantly affect Ca^2+^ homeostasis in the cell and intracellular Ca^2+^ stores by oxidising multiple methionine residues within the Ca^2+^ signalling protein calmodulin (CaM) resulting in an inability to activate a range of target proteins, including the cell membrane Ca-ATPase involved in the maintenance of Ca^2+^ homeostasis [10, 36, 37]. The analysis reported here allowed to clarify how these two mechanisms are interconnected and whether a precise cause-and-effect relationship exists. The kinetics of these processes in neuroblastoma cells and primary rat cortical neurons were analysed under various conditions in which ROS production or Ca^2+^ influx induced by misfolded protein oligomers were specifically inhibited, showing that the lack of an influx of Ca^2+^ ions into the cytosol can reduce the ROS production, whereas the protection against ROS formation did not prevent the initial Ca^2+^ flux but allowed the cells to react, on a longer term, to the initial Ca^2+^ dyshomeostasis, restoring the normal levels of the ions.

## Materials and methods

### Preparation of HypF-N oligomers and Aβ_42_ ADDLs

Wild-type HypF-N from *E. coli* was prepared and purified as described [38], and stored at − 80 °C in 20 mM potassium phosphate buffer, pH 7.0, with 2 mM dithiothreitol (DTT). Type A oligomers (OAs) were prepared by incubating HypF-N at 48 μM, with 50 mM acetate buffer, 12% (*v*/*v*) trifluoroethanol (TFE), 2 mM DTT, pH 5.5, 25 °C, for 4 h without agitation, as previously described [38].

Lyophilised Aβ_42_ (Bachem, Bubendorf, Switzerland) was dissolved in HFIP to 1.0 mM and incubated for 1 h at room temperature to allow complete peptide monomerization. Aβ_42_-derived diffusible ligands (ADDLs) were prepared as described previously [39]. In particular, the HFIP was evaporated with a gentle flow of N_2_ and the dried protein was resuspended to 5 mM with DMSO and then diluted with F-12 HAM to 100 μM. The sample was then incubated at 4 °C for 24 h and centrifuged at 12,000*g* for 10 min to collect the supernatant.

### Cell cultures

Human SH-SY5Y neuroblastoma cells (ATCC CRL-2266, Manassas, VA, USA) were cultured in Dulbecco’s Modified Eagle’s Medium (DMEM), F-12 HAM with 25 mM *N*-2-hydroxyethylpiperazine-*N*-2-ethanesulfonic acid (HEPES) and NaHCO_3_ (1:1) and supplemented with 10% fetal bovine serum (FBS), 2 mM glutamine and 1% antibiotics, as reported [40]. Cell cultures were maintained in a 5% CO_2_ humidified atmosphere at 37 °C and grown until they reached 80% confluence for a maximum of 20 passages. Primary rat cortical neurons (Thermo Fisher Scientific) were plated in 24-well plate at the density of 200,000 cells per well and maintained in neuronal basal plus medium (Thermo Fisher Scientific) supplemented with GlutaMAX (Gibco) at the concentration of 0.5 mM and 2% (*v*/*v*) B-27 serum-free complement (Gibco), in a 5% CO_2_ humidified atmosphere at 37 °C. Every 4 days the medium was partially replaced with fresh one. All the experiments were performed 12–16 days after plating.

### Cell treatments

SH-SY5Y cells were plated in 6-well plates containing coverslips at a density of 40,000 cells per well at 37 °C. After 24 h, they were washed with PBS and treated with HypF-N OAs diluted in cellular medium at the monomer equivalent concentration of 12 µM for 5, 10, 15, 30 and 60 min, or with Aβ_42_ ADDLs diluted in cellular medium at the monomer equivalent concentration of 1 µM for 5, 10, 15, 30, 60, 90, 120 and 180 min. In other sets of experiments, before the treatment with HypF-N OAs or Aβ_42_ ADDLs, SH-SY5Y cells were pre-treated for 1 h with 5 µM CNQX, or 10 µM memantine, or both. In other sets of experiments, cells were pre-treated with 2 µM l-α-lysophosphatidylcholine (LPC) for 2 h, with 30 µM Trolox for 1 h, or in a medium without Ca^2+^ for 1 h. In another set of experiments, the SH-SY5Y cells were treated for 10 min with 1 mM NMDA or 50 µM AMPA, with or without pre-treatment with 30 µM Trolox for 1 h.

Primary rat cortical neurons were plated in 24-well plates containing glass coverslips coated with poly-D-lysine at a density of 200,000 cells per well at 37 °C. 12–16 days after plating, they were washed with PBS and treated with Aβ_42_ ADDLs diluted in cellular medium at the monomer equivalent concentration of 1 µM for 10 and 60 min. In other sets of experiments, before the treatment with Aβ_42_ ADDLs, the cells were pre-treated for 1 h with 5 µM CNQX, or 10 µM memantine, or 30 µM Trolox.

### Measurement of cytosolic free Ca^2+^ levels and intracellular ROS production

Cytosolic Ca^2+^ levels were measured in living SH-SY5Y cells and primary rat cortical neurons after the different treatments, or after adding 1 µM ionomycin for 1 h as a positive control. The cells were then washed with PBS and loaded with 4 µM Fluo-4 AM (Thermo Fisher Scientific) for 10 min. The Ca^2+^ levels for untreated cells were evaluated in SH-SY5Y cells at each time point, from 0 to 180 min, changing the cellular medium at the different time lengths, washing with PBS and loading the Fluo-4 AM probe for 10 min. Considering the absence of changes in basal Ca^2+^ levels, all data are reported relative to untreated cells at time 0. ROS levels were measured in living SH-SY5Y cells and primary rat cortical neurons after the different treatments, or after adding 250 µM H_2_O_2_ for 1 h, as a positive control, and then by loading 5 µM 5-(and-6)-chloromethyl-2′,7′-dichlorodihydrofluorescein diacetate (CM-H_2_DCFDA) in the last 15 min of the different treatments. ROS levels for untreated cells were evaluated in SH-SY5Y cells at the different time points, changing the cell medium and loading the probe in the last 15 min of the treatment. Considering the absence of significant variation, all data are reported relative to untreated cells at 15 min, which is the probe incubation time and therefore the shortest time that can be analysed. Both Ca^2+^ and ROS levels were then evaluated after excitation at 488 nm by a TCS SP8 scanning confocal microscopy system equipped with an argon laser source (Leica Microsystems).

In another set of experiments, cytosolic Ca^2+^ and ROS levels were measured in living SH-SY5Y cells by loading 5 µM X-Rhod-1 AM (Thermo Fisher Scientific) in the last 20 min and 5 µM CellRox™ Deep Red Reagent (Thermo Fisher Scientific) in the last 30 min of the different treatments, respectively. Ca^2+^ and ROS levels were then evaluated after excitation at 561 and 633 nm, respectively, by the same TCS SP8 scanning confocal microscopy system described above.

In all cases, a series of 1 µm thick optical sections (1024 × 1024) was taken through the cell depth for each sample using a Leica Plan Apo 63 × oil immersion objective and projected as a single composite image by superimposition (Leica Microsystems). Three different experiments were carried out and 10–22 cells were analysed in each experiment, in both Ca^2+^ and ROS analyses, using Image J software. Values were averaged over the 10–22 cells in each experiment and the mean and standard error of the mean (SEM) were determined from the averaged values of the three experiments (*n* = 3). All data were normalized to the positive control value, obtained with ionomycin and H_2_O_2_ respectively, which were attributed 100%.

### Statistical analysis

All data were expressed as means ± SEM (standard error of the mean). Comparisons between the different groups were performed by Student’s t-test. The single (*;§), double (**;§§) and triple (***;§§§) symbols refer to *p* values < 0.05, < 0.01 and < 0.001, respectively.

## Results

### Toxic HypF-N oligomers increase intracellular Ca^2+^ levels and ROS production

We started our analysis using model oligomers formed by the protein HypF-N (named type A oligomers, or OAs), which were previously found to have effects similar to those of Aβ_42_ oligomers in cell and animal models [10, 11, 38, 41, 42]. These HypF-N OAs are highly stable, versatile, easy to isolate and have a non-toxic counterpart (known as type B oligomers, or OBs), which is useful as a negative control [38].

**Fig. 1:**
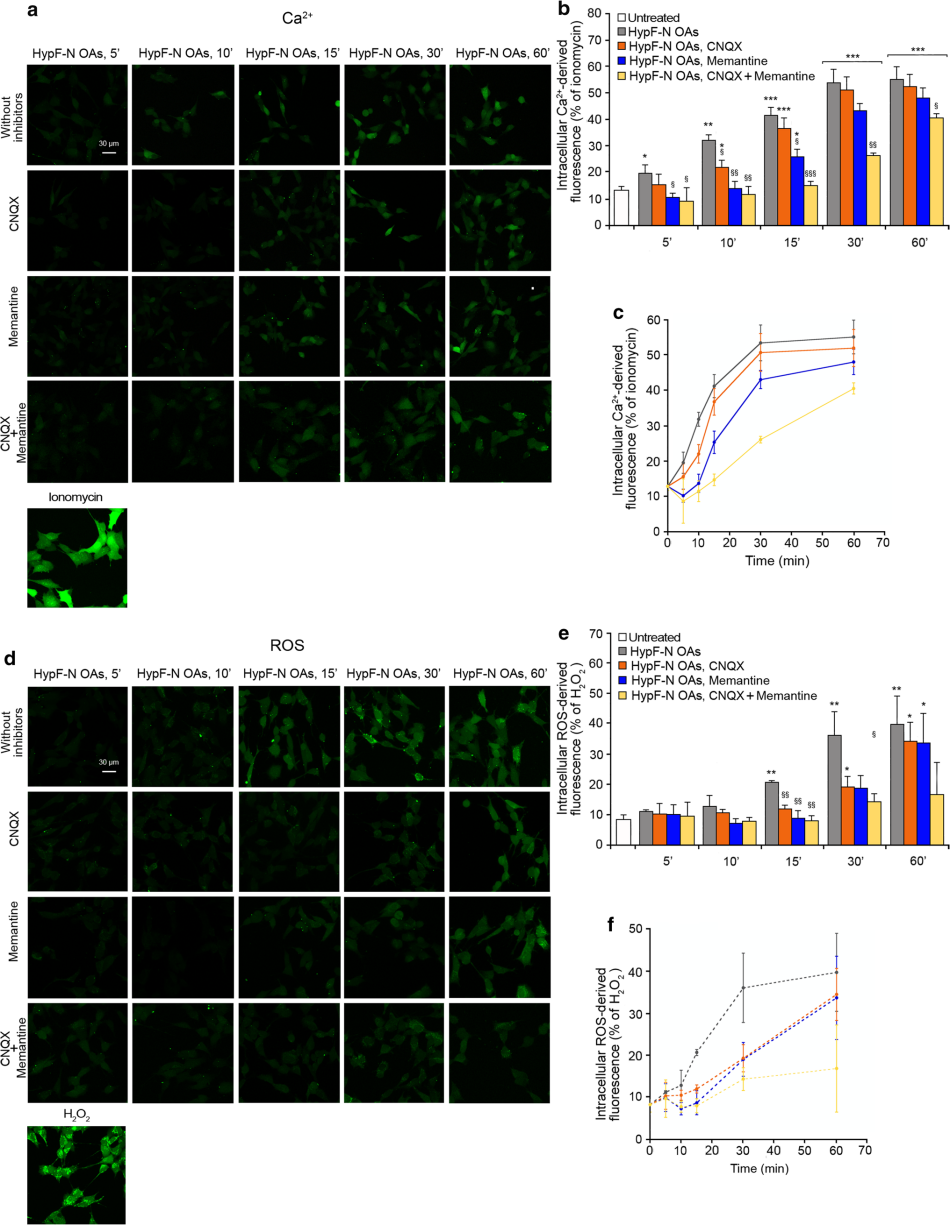
Toxic HypF-N oligomers increase intracellular Ca^2+^ levels and ROS production. **a** Representative confocal scanning microscopy images of free Ca^2+^ levels in SH-SY5Y cells following the treatment with no inhibitors (first row), 5 µM CNQX (second row), 10 µM memantine (third row), and both inhibitors (fourth row), and analysed after 5, 10, 15, 30 and 60 min of treatment with 12 µM (monomer equivalents) HypF-N OAs. A positive control of Ca^2+^ influx following treatment with 1 µM ionomycin for 1 h is shown at the bottom. **b** Semi-quantitative analysis of intracellular free Ca^2+^-derived fluorescence. The Ca^2+^ levels for untreated cells were not found to vary with time (Fig. S1a–c) and for simplicity the value recorded at time 0 min was reported, here and in other figures. **c** Kinetic plots showing the fluorescence *versus* time as reported in panel **b**. **d** Representative confocal scanning microscopy images of intracellular ROS levels in SH-SY5Y cells following the treatment with no inhibitors (first row), 5 µM CNQX (second row) 10 µM memantine (third row) and both inhibitors (fourth row), and analysed after 5, 10, 15, 30 and 60 min of treatment with 12 µM (monomer equivalents) HypF-N OAs. A positive control of ROS production following treatment with 250 µM H_2_O_2_ for 1 h is shown at the bottom. **e** Semi-quantitative analysis of intracellular ROS-derived fluorescence. The ROS levels for untreated cells were not found to vary with time (Fig. S1d–f) and for simplicity the value recorded at time 15 min was reported, here and in other figures. **f** Kinetic plots showing the fluorescence *versus* time as reported in panel **e**. Three different experiments were carried out, with 10–22 cells each, for each condition. Data are represented as mean ± SEM (n = 3). The single (*), double (**) and triple (***) asterisks refer to *p* values < 0.05, < 0.01 and < 0.001, respectively, relative to untreated cells. The single (§), double (§§) and triple (§§§) symbols refer to *p* values < 0.05, < 0.01 and < 0.001, respectively, relative to HypF-N OAs without inhibitors at corresponding time points

In a previous work it was shown that the HypF-N OAs, similarly to the Aβ_42_ ADDLs, are able to cause a progressive increase of intracellular Ca^2+^ levels on SH-SY5Y cells by activating rapidly extrasynaptic NMDA receptors and, to a lower extent, AMPA receptors [14]. We therefore started our analysis by evaluating whether the freshly formed toxic HypF-N OAs prepared for this study confirmed this effect. The treatment over time of SH-SY5Y cells with HypF-N OAs (12 μM monomer equivalents) showed a gradual increase of intracellular Ca^2+^ levels, which was evident already after 5 min and reached the maximum level after 30 min of treatment (images in Fig. 1a, histograms in Fig. 1b and corresponding kinetic plot in Fig. 1c). When cells were pre-treated with CNQX, an AMPA receptor competitive antagonist, or with memantine, a NMDA receptor uncompetitive inhibitor, a slight reduction of the OA-induced cytoplasmic Ca^2+^ increase was observed in the early stages, up to 10 min of treatment, which was more significant with memantine (Fig. 1a–c). This reduction was followed by a gradual increase of the intracellular Ca^2+^ concentration, until normal levels reached after 60 min of treatment (Fig. 1a–c). Overall, these pre-treatments cause a deceleration of the increase of intracellular Ca^2+^ (Fig. 1c). Moreover, after a pre-treatment with both CNQX and memantine, the intracellular Ca^2+^ levels remained similar to untreated cells up to 15 min of treatment, and significantly lower than those of cells without inhibitor pre-treatment up to 60 min of oligomer treatment (Fig. 1a,b), further slowing down the kinetics of OA-induced Ca^2+^ increase (Fig. 1c).

We then focused our attention on ROS production in SH-SY5Y cells with the same conditions of oligomer treatment and inhibitor pre-treatment described above. The treatment with HypF-N OAs (12 μM monomer equivalents) showed a slow and gradual increase of ROS production, which was slower than that observed by monitoring Ca^2+^ concentration, clearly detectable after 15 min, and reaching the maximum level after 30–60 min of treatment (images in Fig. 1d, histograms in Fig. 1e and corresponding kinetic plot in Fig. 1f). Cellular pre-treatment with CNQX or memantine determined again a reduction of cytoplasmic ROS levels in the early stages, up to 30 min of oligomer treatment, followed by an increase, until almost normal levels were reached after 60 min of treatment (Fig. 1d,e). Overall, these pre-treatments caused a deceleration of the increase of ROS levels (Fig. 1f), which was even more marked than that detected by monitoring intracellular Ca^2+^ levels. Moreover, after a pre-treatment with both CNQX and memantine, the ROS levels remained significantly lower than those of cells without inhibitor pre-treatment, up to 60 min of oligomer treatment (Fig. 1d,e).

**Fig. 2:**
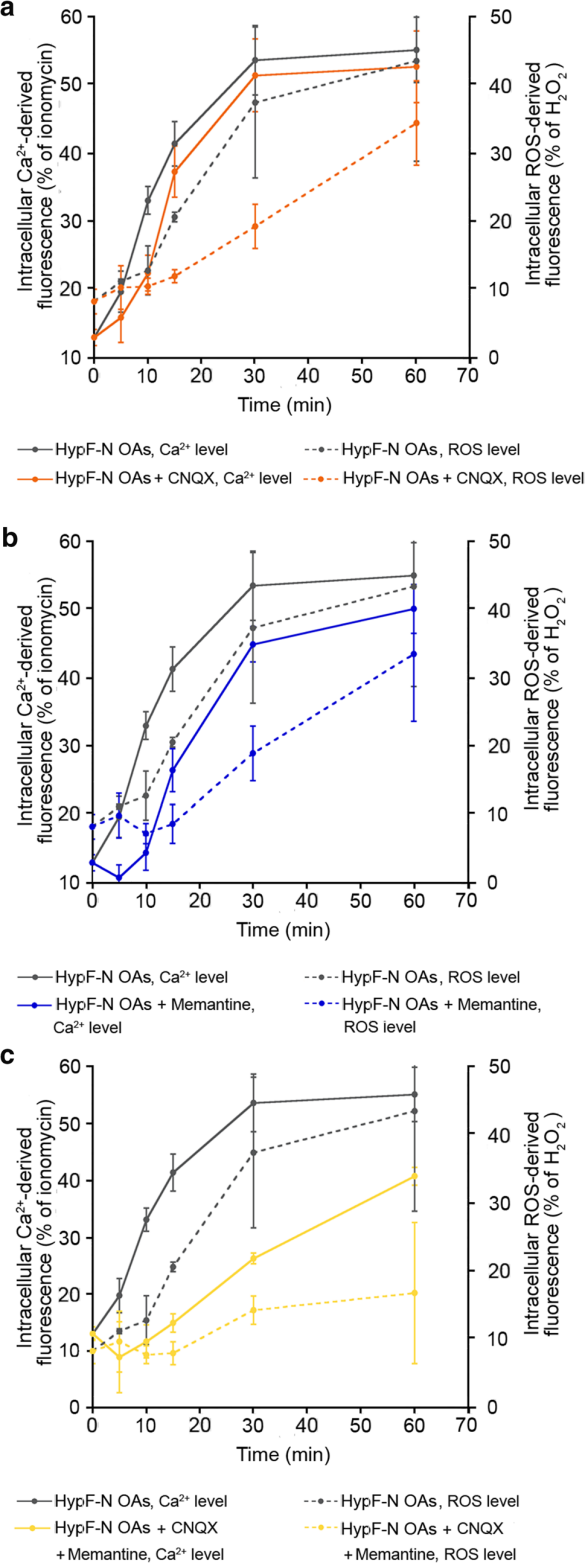
Increase of intracellular Ca^2+^ levels precedes ROS production. **a**–**c** Kinetic plots showing the fluorescence associated with intracellular Ca^2+^ and ROS *versus* time after treatment with HypF-N OAs. The time courses refer to Ca^2+^ levels (solid lines) and ROS levels (dotted lines) without inhibitors (grey), with CNQX (orange), with memantine (blue) and with both CNQX and memantine (yellow)

Comparing the kinetics of cytosolic Ca^2+^ and ROS increases induced by HypF-N OAs in the absence of NMDA/AMPA inhibitors, the ROS time course appears to be slower, particularly in the first minutes (Fig. 2a). A lag time appears to be present only in the ROS time course, suggesting that the increase of ROS levels follows that in Ca^2+^ (Fig. 2a). Comparing the times courses in the presence of CNQX, memantine, or both, the ROS time course appears again to be slower than the corresponding time course of Ca^2+^ (Fig. 2a–c). Interestingly, we also observed a longer delay in ROS level increase following pre-treatment with CNQX (Fig. 2a, orange dotted line), or memantine (Fig. 2b, blue dotted line) or both (Fig. 2c, yellow dotted line) compared to Ca^2+^ levels following the same pre-treatment (Fig. 2a–c, orange, blue and yellow line, respectively), suggesting that inhibition of AMPA and NMDA receptors, with the consequent reduction of the early Ca^2+^ influx, allowed the cells to postpone the production of ROS even more markedly. Overall, all the kinetic data indicate that an increase of intracellular Ca^2+^ levels precedes ROS production.

### Lysophosphatidylcholine enrichment reduces both Ca^2+^ level increase and ROS production

**Fig. 3:**
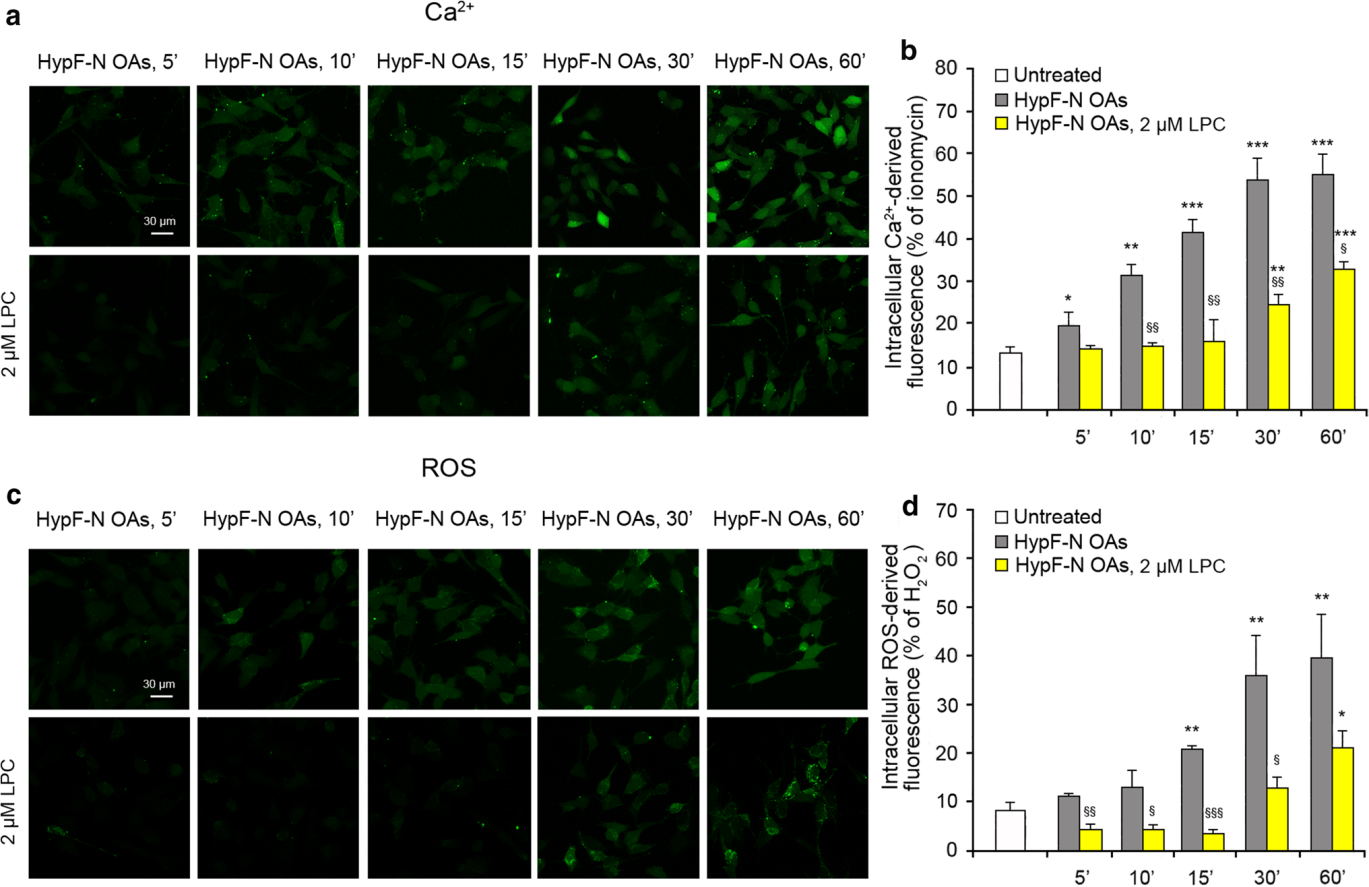
Lysophosphatidylcholine (LPC) enrichment reduces both the Ca^2+^ level increase and ROS production. **a** Representative confocal scanning microscopy images of intracellular free Ca^2+^ levels in SH-SY5Y cells following no treatment (first row) and treatment with 2 µM LPC (second row), and analysed after 5, 10, 15, 30, and 60 min of treatment with 12 µM (monomer equivalents) HypF-N OAs. **b** Semi-quantitative analysis of intracellular Ca^2+^-derived fluorescence. The value for untreated cells refers to 0 min and did not change with time. **c** Representative confocal scanning microscopy images of intracellular ROS levels in SH-SY5Y cells following no treatment (first row) and treatment with 2 µM LPC (second row), and analysed after 5, 10, 15, 30, and 60 min of treatment with 12 µM (monomer equivalents) HypF-N OAs. **d** Semi-quantitative analysis of intracellular ROS-derived fluorescence. The value for untreated cells refers to 15 min and did not change with time. Three different experiments were carried out, with 10–22 cells each, for each condition. Data are represented as mean ± SEM (*n* = 3). The single (*), double (**) and triple (***) asterisks refer to *p* values < 0.05, < 0.01 and < 0.001, respectively, relative to untreated cells. The single (§), double (§§) and triple (§§§) symbols refer to *p* values < 0.05, < 0.01 and 0.001, respectively, relative to HypF-N OAs without treatment with LPC at corresponding time points

It was previously shown that the enrichment of SH-SY5Y cell membranes with 2 μM lysophosphatidylcholine (LPC) inhibits the OA-induced Ca^2+^ flow mediated by the mechanosensitive NMDA receptors, suggesting that the opposing force exerted by LPC (compression) effectively inhibits the mechanical signal (stretching) generated by the action of the oligomers onto the membrane [14]. To investigate whether this inhibition is also effective on ROS production, we pre-treated SH-SY5Y cells with 2 μM LPC for 2 h, and then we treated them with HypF-N OAs (12 μM monomer equivalents) for 5, 10, 15, 30 and 60 min, monitoring both intracellular Ca^2+^ and ROS levels. In the presence of LPC, the Ca^2+^ levels remained similar to those of untreated cells up to 15 min; they then increased, but remained significantly lower than those recorded without LPC at corresponding time points, even after 60 min of treatment (Fig. 3a,b).

The ROS levels also remained constant for 15 min and then increased, but remained significantly lower than the corresponding values in the absence of LPC pre-treatment, up to 60 min (Fig. 3c,d). These two time courses suggest that the LPC-mediated membrane compression is able to inhibit the OA-induced Ca^2+^ flow through NMDA receptors for a prolonged time and the subsequent rise of ROS levels. They also confirm the presence of an interconnection between the two mechanisms.

### Intracellular Ca^2+^ influx and ROS production induced by HypF-N OAs are connected

As observed in the previous experiments, the intracellular rise of free Ca^2+^ is associated with the elevation of ROS following treatment with HypF-N OAs. The kinetic traces described so far lead to the hypothesis that the second event is caused by the first, rather than being independent of it, based on the observation that: (i) the former is more rapid than the latter, (ii) the rise of ROS has a lag time and follows the lag-independent rise of Ca^2+^, (iii) the lag times are longer in the ROS time courses than in the corresponding Ca^2+^ time courses in the presence of NMDA/AMPA inhibitors, and (iv) the effects caused by inhibitors of the Ca^2+^ flow (CNQX and memantine) are even larger on the time-dependent increase of ROS than Ca^2+^.

**Fig. 4:**
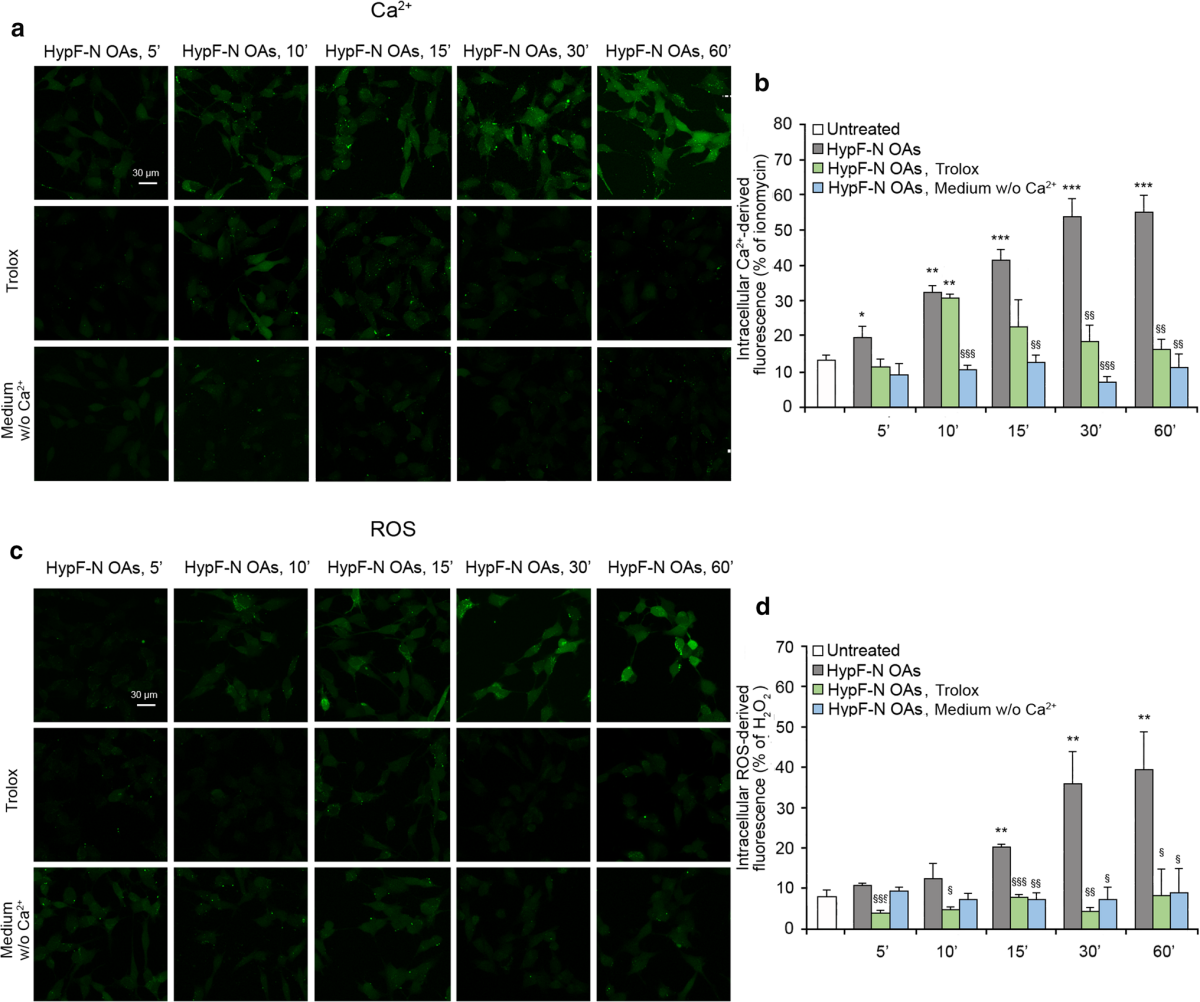
Intracellular Ca^2+^ influx and ROS production induced by HypF-N OAs are connected. **a** Representative confocal scanning microscopy images of intracellular free Ca^2+^ levels in SH-SY5Y cells following no treatment (first row), pre-treatment with 30 µM Trolox (second row), and in a medium without Ca^2+^ (third row), and analysed after 5, 10, 15, 30, and 60 min of treatment with 12 µM (monomer equivalents) HypF-N OAs. **b** Semi-quantitative analysis of intracellular Ca^2+^-derived fluorescence. The value for untreated cells refers to 0 min and did not change with time. **c** Representative confocal scanning microscopy images of intracellular ROS levels in SH-SY5Y cells following no treatment (first row), pre-treatment with 30 µM Trolox (second row), and in a medium without Ca^2+^ (third row), and analysed after 5, 10, 15, 30, and 60 min of treatment with 12 µM (monomer equivalents) HypF-N OAs. **d** Semi-quantitative analysis of intracellular ROS-derived fluorescence. The value for untreated cells refers to 15 min and did not change with time. Three different experiments were carried out, with 10–22 cells each, for each condition. Data are represented as mean ± SEM (*n* = 3). The single (*), double (**) and triple (***) asterisks refer to p values < 0.05, < 0.01 and < 0.001, respectively, relative to untreated cells. The single (§), double (§§) and triple (§§§) symbols refer to *p* values < 0.05, < 0.01 and 0.001, respectively, relative to HypF-N OAs without treatment with Trolox or Ca^2+^-deprived medium at corresponding time points

Since these suggestions are only kinetic and, therefore, not definitive, we further investigated if the two processes are linked to each other in our system with a clear cause-and-effect relationship between them. For this purpose, we treated the SH-SY5Y cells with HypF-N OAs over time, after a 1 h pre-treatment with 30 µM Trolox, which is a highly soluble and membrane-unbound antioxidant analogue of Tocopherol [43]. An increase of cytosolic Ca^2+^ concentration was observed in the early stages up 10 min, followed by a reduction down to levels observed in untreated cells (Fig. 4a,b, green bars). By contrast, the levels of ROS did not increase and were similar to the untreated cells at all time points, up to 60 min of OAs treatment (Fig. 4c,d, green bars). These results indicate that Trolox acted correctly as an antioxidant preventing ROS production in cells very effectively within the time frame explored here, but it did not prevent the early rise of Ca^2+^ mediated by AMPA and NMDA receptors. However, the presence of the antioxidant in the medium allowed the cells to re-establish the normal Ca^2+^ homeostasis that had been initially lost as a consequence of the activation of NMDA and AMPA receptors, indicating that the lack of ROS production allows the cells to face effectively the stress induced by the HypF-N OAs and the Ca^2+^ flow across the cell membrane.

In a control experiment, to assess whether Trolox interferes directly with the AMPA and NMDA receptor opening, we activated the two receptors using their specific agonists, AMPA and NMDA, respectively, after a 1 h pre-treatment with 30 µM Trolox, finding that both agonists are able to induce an increase of the intracellular Ca^2+^ levels independently of the presence of the antioxidant agent (Fig. S2).

With the same purpose of investigating the cause-and-effect link between Ca^2+^ and ROS level increases, we treated the SH-SY5Y cells with HypF-N OAs over time, in a Ca^2+^-free medium (Fig. 4, light blue bars). In this case, the OA-induced increase of cytosolic Ca^2+^ was fully inhibited, up to 60 min (Fig. 4a,b, light blue bars), confirming previous demonstrations that the source of such intracellular Ca^2+^ ions is the extracellular medium rather than intracellular organelles [10]. It is interesting to note that a complete inhibition of ROS production was also observed, again up to 60 min (Fig. 4c,d, light blue bars).

Taken together, the kinetic data obtained with Trolox and the Ca^2+^-free medium indicate that the lack of an influx of Ca^2+^ ions from the extracellular space into the cytosol is able to reduce ROS production, whereas the protection against ROS formation does not prevent an initial rise of intracellular Ca^2+^ concentration, underling the consequential nature of ROS formation relative to Ca^2+^ influx. They also provide evidence on the existence of an oxidative metabolism required to restore Ca^2+^ homeostasis and responsible for ROS accumulation, which does not allow an effective pumping of Ca^2+^ ions outside the cells, unless an antioxidant environment maintains the levels of ROS under control, allowing the cells to restore Ca^2+^ homeostasis effectively (see “Discussion” for further details).

### Aβ_42_ ADDLs oligomers increase intracellular Ca^2+^ levels and ROS production

**Fig. 5:**
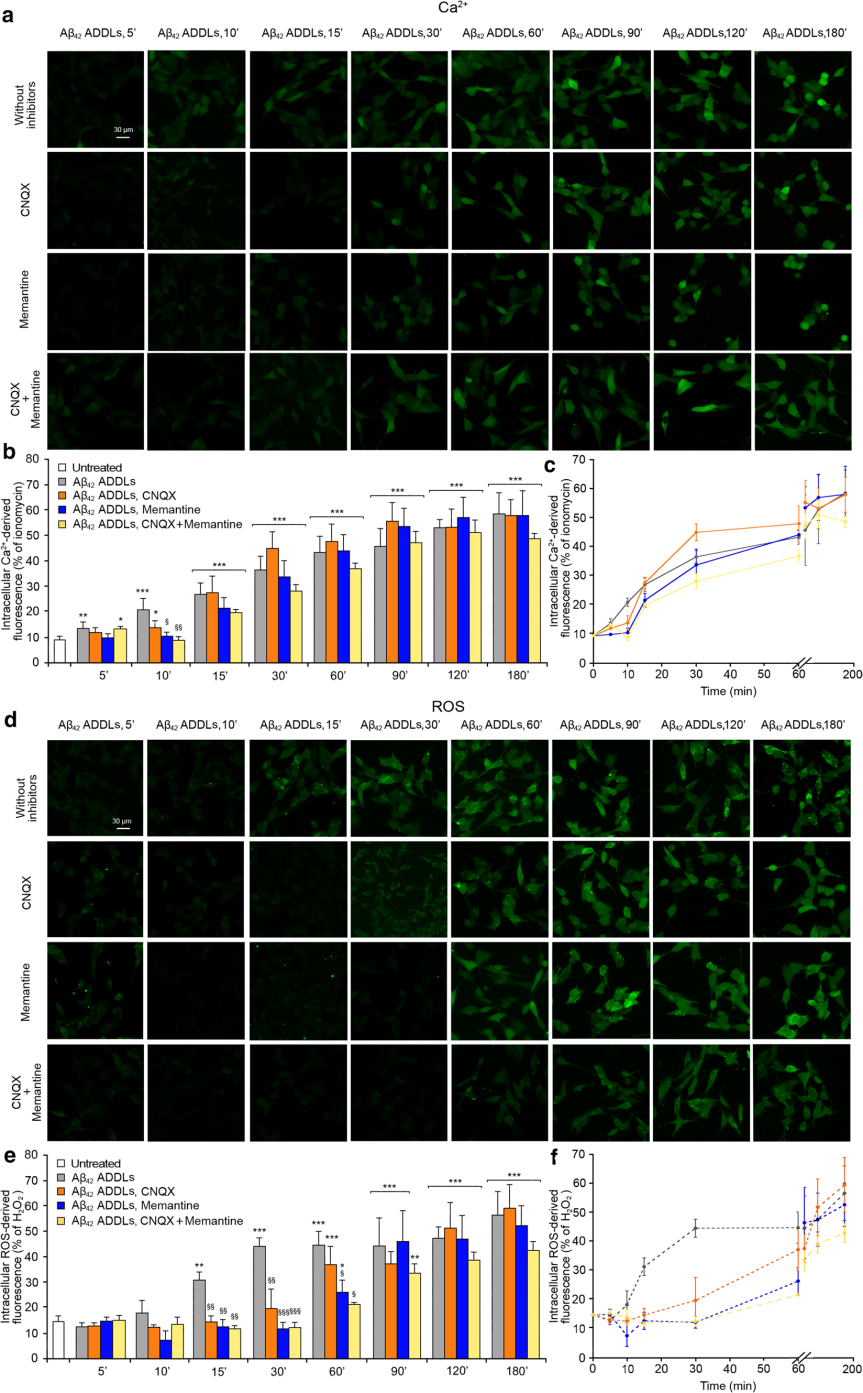
Aβ_42_ ADDLs oligomers increase intracellular Ca^2+^ levels and ROS production in SH-SY5Y cells. **a** Representative confocal scanning microscopy images of free Ca^2+^ levels in SH-SY5Y cells following the treatment with no inhibitors (first row), 5 µM CNQX (second row), 10 µM memantine (third row), and both inhibitors (fourth row), and analysed after 5, 10, 15, 30, 60, 90, 120 and 180 min of treatment with 1 µM (monomer equivalents) Aβ_42_ ADDLs oligomers. **b** Semi-quantitative analysis of intracellular free Ca^2+^-derived fluorescence. The value for untreated cells refers to 0 min and did not change with time. **c** Kinetic plots showing the fluorescence *versus* time as reported in panel **b**. **d** Representative confocal scanning microscopy images of intracellular ROS levels in SH-SY5Y cells following the treatment with no inhibitors (first row), 5 µM CNQX (second row), 10 µM memantine (third row), and both inhibitors (fourth row), and analysed after 5, 10, 15, 30, 60, 90, 120 and 180 min of treatment with 1 µM (monomer equivalents) Aβ_42_ ADDLs oligomers. **e** Semi-quantitative analysis of intracellular ROS-derived fluorescence. The value for untreated cells refers to 15 min and did not change with time. **f** Kinetic plots showing the fluorescence *versus* time as reported in panel **e**. Three different experiments were carried out, with 10–22 cells each, for each condition. Data are represented as mean ± SEM (*n* = 3). The single (*), double (**) and triple (***) asterisks refer to *p* values < 0.05, < 0.01 and < 0.001, respectively, relative to untreated cells. The single (§), double (§§) and triple (§§§) symbols refer to *p* values < 0.05, < 0.01 and 0.001, respectively, relative to Aβ_42_ ADDLs oligomers without inhibitors at corresponding time points

We then extended the analysis carried out with the model HypF-N OAs to Aβ oligomers, using Aβ_42_-derived diffusible ligands (Aβ_42_ ADDLs) [39] at the concentration of 1 μM. In previous works it was shown that Aβ_42_ ADDLs, similarly to HypF-N OAs, are able to cause a progressive increase of the intracellular Ca^2+^ levels in SH-SY5Y cells by activating rapidly extrasynaptic NMDA and AMPA receptors [14]. We therefore prepared freshly formed Aβ_42_ ADDLs oligomers and evaluated whether they maintained this effect. The treatment over time of SH-SY5Y cells with Aβ_42_ ADDLs showed a gradual increase of the intracellular Ca^2+^ levels, which was clearly detectable already after 5 min and reached a plateau after 180 min of treatment (images in Fig. 5a, histograms in Fig. 5b and corresponding kinetic plot in Fig. 5c). When cells were pre-treated with CNQX, with memantine, or with both CNQX and memantine, a slight reduction of the Aβ_42_ ADDLs-induced cytoplasmic Ca^2+^ increase was observed in the early stages, up to 10 min of treatment (Fig. 5a,b). With Aβ_42_ ADDLs, a combination of both inhibitors showed kinetics similar to the memantine treatment. This reduction was followed by a gradual increase of the intracellular Ca^2+^ concentration, until normal levels were reached after prolonged treatment (Fig. 5a,b). Overall, these pre-treatments cause a deceleration of the intracellular Ca^2+^ increase at early time points (Fig. 5c).

The treatment of SH-SY5Y cells with Aβ_42_ ADDLs under the same conditions also showed a gradual increase of ROS production, which was evident after 15 min up to 180 min, and hence slower than that observed by monitoring Ca^2+^ concentration (Fig. 5d–f). Interestingly, such increase appeared to occur more rapidly than that observed with HypF-N OAs, which can be attributed to the known oxidative potential of Aβ_42_ ADDLs through Ca^2+^-independent mechanisms [44–46]. Cellular pre-treatment with CNQX or memantine, or both inhibitors, determined again a reduction of ROS levels in the early stages, up to 30 min for CNQX and 60 min for memantine and both inhibitors together, followed by a gradual increase, until normal levels were reached after 90 min (Fig. 5d–f). These pre-treatments, therefore, caused a deceleration of the ROS increase mediated by the oligomers (Fig. 5f), which was again more marked than that detected by monitoring intracellular Ca^2+^ levels. All these results confirmed the observation with the HypF-N OAs.

**Fig. 6:**
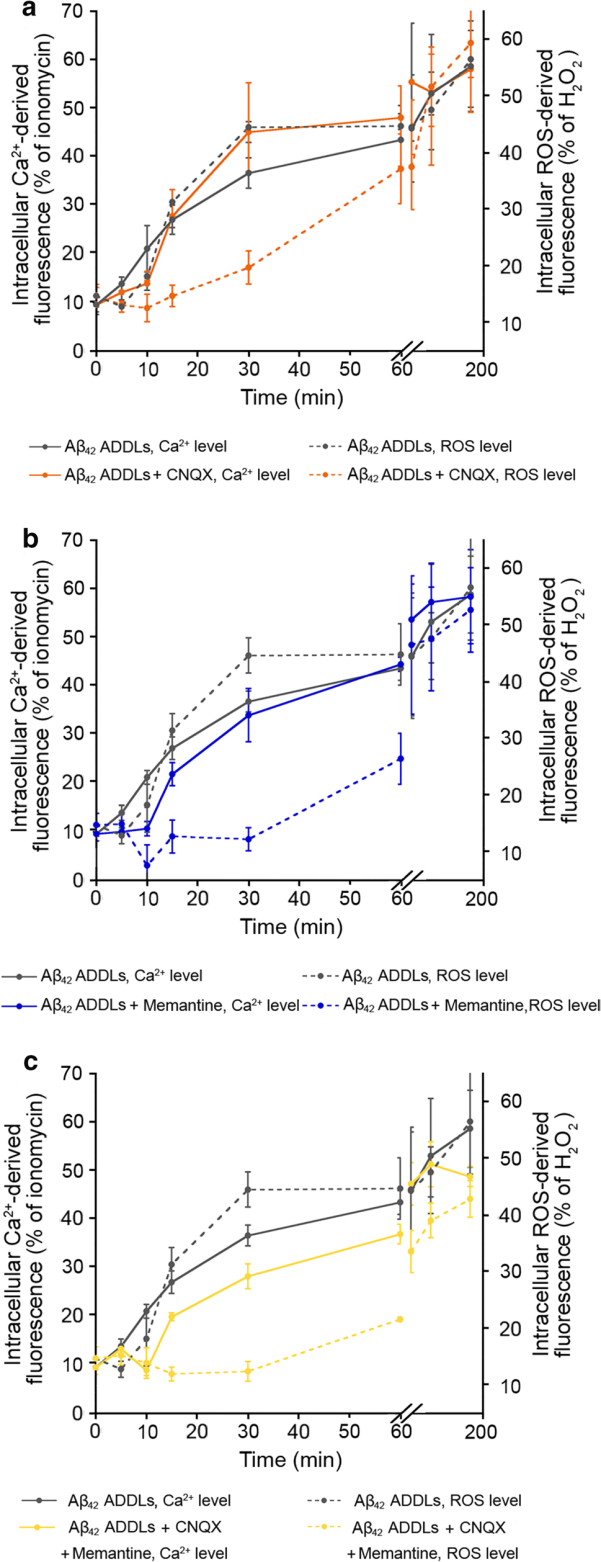
Increase of intracellular Ca^2+^ levels anticipates ROS production in SH-SY5Y cells. **a–c** Kinetic plots showing the fluorescence associated with intracellular Ca^2+^ and ROS *versus* time after treatment with Aβ_42_ ADDLs. The time courses refer to Ca.^2+^ levels (solid lines) and ROS levels (dotted line) without inhibitors (grey), with CNQX (orange), with memantine (blue) and with both CNQX and memantine (yellow)

Comparing the Ca^2+^ and ROS kinetics without NMDA/AMPA inhibitors, the ROS time course appears to be slower in the first minutes (Fig. 6a), suggesting that the increase of the intracellular Ca^2+^ levels anticipates ROS production. Moreover, comparing the times courses in the presence of CNQX or memantine, or both, the ROS time courses appear again to be slower than the corresponding time courses of Ca^2+^ (Fig. 6a–c). Also with this type of oligomers we observed a longer delay in ROS level increase following pre-treatment with CNQX (Fig. 6a, orange dotted line) or memantine (Fig. 6b, blue dotted line) or both (Fig. 6c, yellow dotted line), compared to the Ca^2+^ kinetics following the same pre-treatment (Fig. 6a,b, orange, blue and yellow line, respectively), confirming that the reduction of the early Ca^2+^ influx, observed by inhibiting the NMDA and AMPA receptors, allowed the cells to delay the production of ROS.

**Fig. 7:**
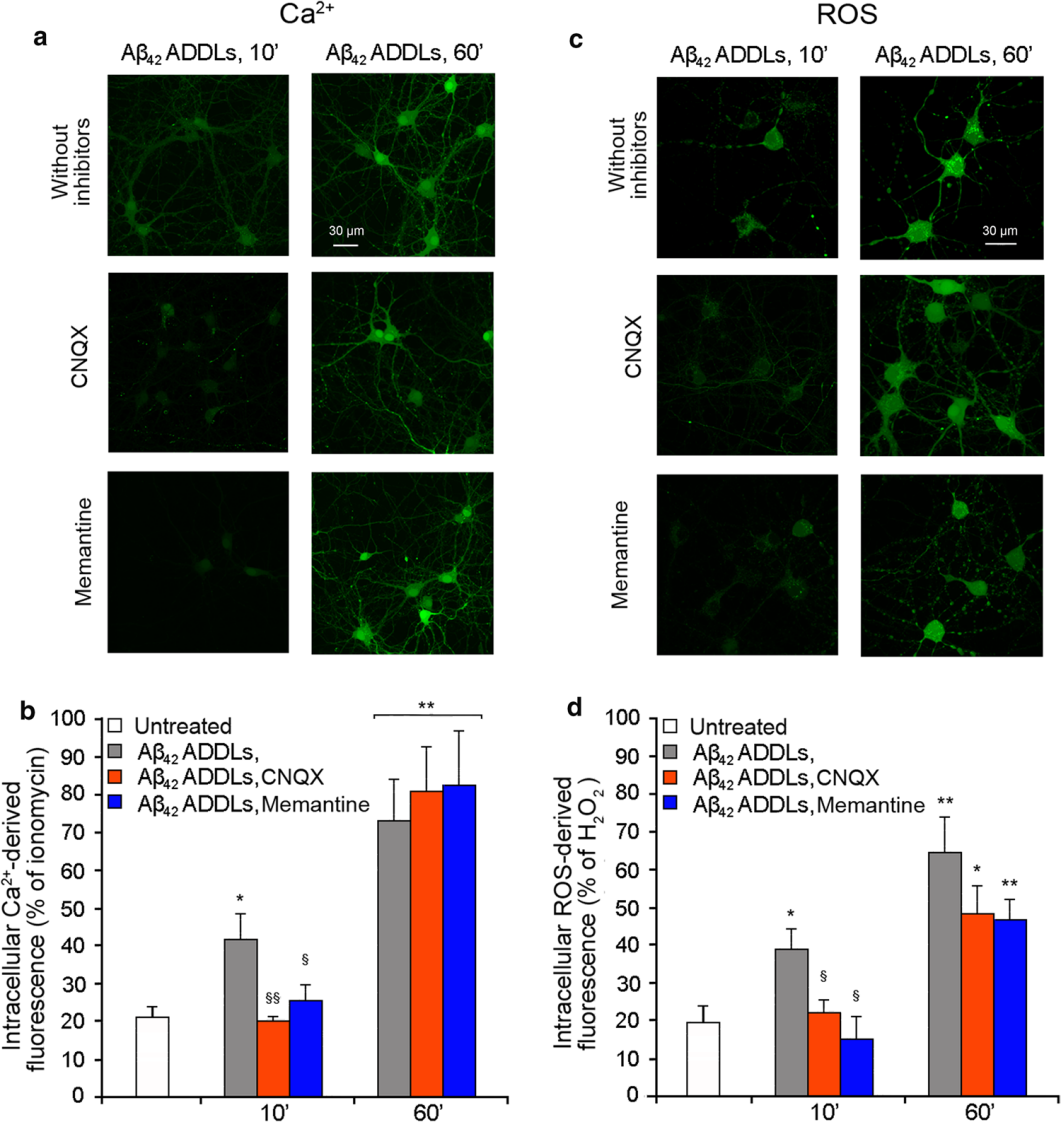
Aβ_42_ ADDLs oligomers increase intracellular Ca^2+^ levels and ROS production in primary rat cortical neurons. **a** Representative confocal scanning microscopy images of intracellular free Ca^2+^ levels in primary rat cortical neurons treated with no inhibitors (first row), 5 µM CNQX (second row) and 10 µM memantine (third row), and analysed after 10 and 60 min of treatment with 1 µM (monomer equivalents) Aβ_42_ ADDLs oligomers. **b** Semi-quantitative analysis of intracellular free Ca^2+^-derived fluorescence. **c** Representative confocal scanning microscopy images of intracellular ROS levels in primary rat cortical neurons treated with no inhibitors (first row), 5 µM CNQX (second row) and 10 µM memantine (third row), and analysed after 10 and 60 min of treatment with 1 µM (monomer equivalents) Aβ_42_ ADDLs oligomers. **d** Semi-quantitative analysis of intracellular ROS-derived fluorescence. Three different experiments were carried out, with 10–22 cells each, for each condition. Data are represented as mean ± SEM (*n* = 3). The single (*) and double (**) asterisks refer to *p* values < 0.05 and < 0.01, respectively, relative to untreated cells. The single (§) and double (§§) symbols refer to *p* values < 0.05 and < 0.01, respectively, relative to Aβ_42_ ADDLs oligomers without inhibitors at corresponding time points

When repeated on primary rat cortical neurons, the Aβ_42_ ADDLs had a similar effect. After 10 min of treatment, the Aβ_42_ ADDLs induced an increase of the intracellular Ca^2+^ levels, which further increased after 60 min of treatment (Fig. 7a,b). When the cells were pre-treated with CNQX or memantine, a significant reduction of the Aβ_42_ ADDLs-induced cytoplasmic Ca^2+^ levels was observed after 10 min of treatment with the oligomers, confirming the involvement of the receptors in the Ca^2+^ influx (Fig. 7a,b). After 60 min of treatment with ADDLs, the levels of Ca^2+^ in the presence of pre-treatment went back to the levels observed in its absence (Fig. 7a,b). Moreover, Aβ_42_ ADDLs also induced an increase of ROS levels after 10 min and a further increase after 60 min of treatment (Fig. 7c,d), with the former being significantly reduced with CNQX or memantine (Fig. 7c,d).

### Intracellular Ca^2+^ influx and ROS production induced by Aβ_42_ ADDLs are connected

**Fig. 8:**
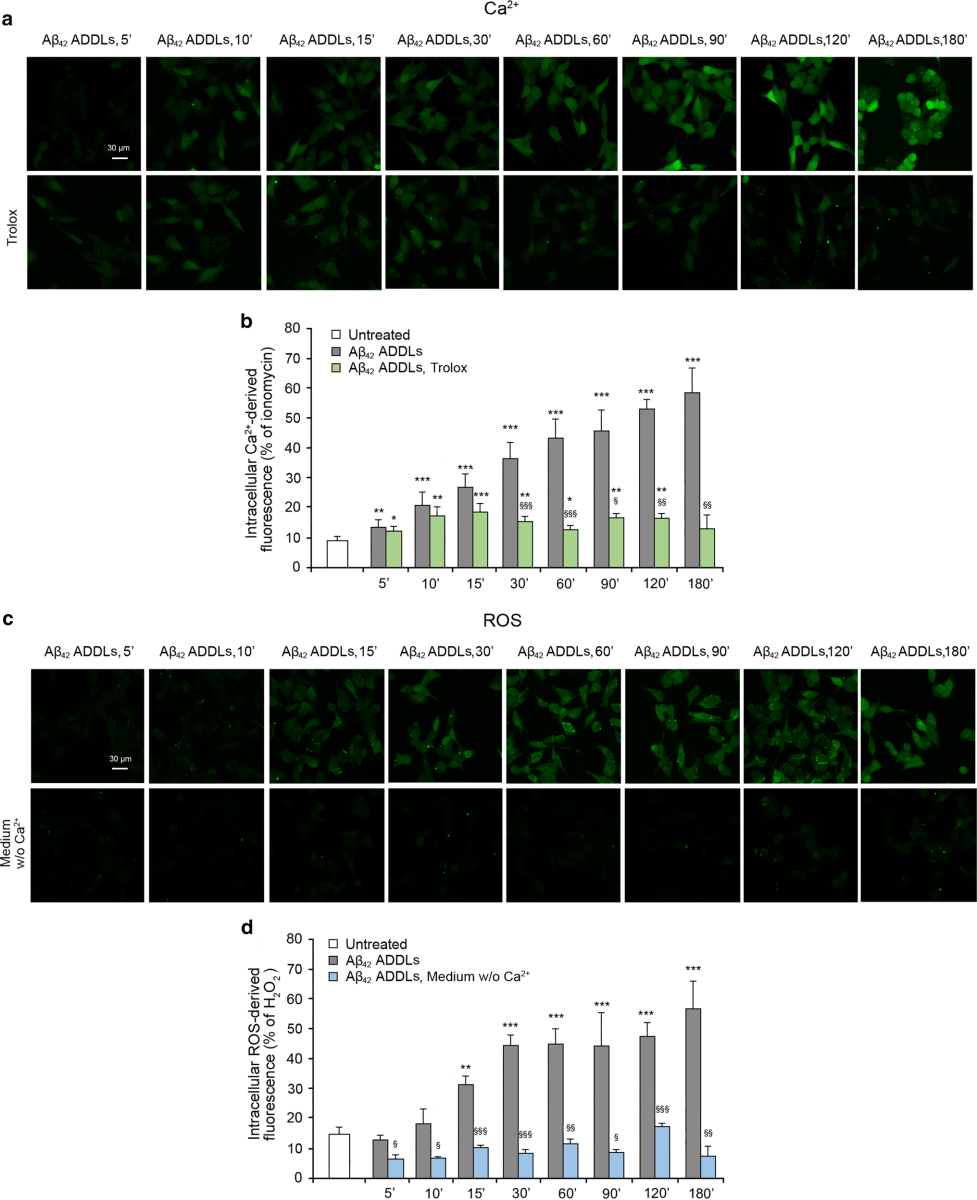
Intracellular Ca^2+^ influx and ROS production induced by Aβ_42_ ADDLs are connected in SH-SY5Y cells. **a** Representative confocal scanning microscopy images of intracellular free Ca^2+^ levels in SH-SY5Y cells following no treatment (first row), and pre-treatment with 30 µM Trolox (second row), and analysed after 5, 10, 15, 30, 60, 90, 120 and 180 min of treatment with 1 µM (monomer equivalents) Aβ_42_ ADDLs oligomers. **b** Semi-quantitative analysis of intracellular Ca^2+^-derived fluorescence. The value for untreated cells refers to 0 min and did not change with time. **c** Representative confocal scanning microscopy images of intracellular ROS levels in SH-SY5Y cells following no treatment (first row), and treatment in a medium without Ca^2+^ (second row), and analysed after 5, 10, 15, 30, 60, 90, 120 and 180 min of treatment with 1 µM (monomer equivalents) Aβ_42_ ADDLs oligomers. **d** Semi-quantitative analysis of intracellular ROS-derived fluorescence. The value for untreated cells refers to 15 min and did not change with time. Three different experiments were carried out, with 10–22 cells each, for each condition. Data are represented as mean ± SEM (*n* = 3). The double (**) and triple (***) asterisks refer to *p* values < 0.01 and < 0.001, respectively, relative to untreated cells. The single (§), double (§§) and triple (§§§) symbols refer to *p* values < 0.05, < 0.01 and < 0.001, respectively, relative to Aβ_42_ ADDLs oligomers without treatment with Trolox or Ca^2+^-deprived medium at corresponding time points

We then treated SH-SY5Y cells with Aβ_42_ ADDLs in the presence and absence of a pre-treatment for 1 h with the antioxidant Trolox. In the presence of Trolox, an initial increase of cytosolic Ca^2+^ concentration was observed, particularly after 10–30 min of treatment with the oligomers, followed by a reduction at180 min (Fig. 8a,b). These results confirm that the maintenance of a redox balance allowed the cells to react to the initial Ca^2+^ flux induced by the Aβ_42_ ADDLs and normalize Ca^2+^ homeostasis, initially lost because of the action of the oligomers.

With the same purpose, ROS production in SH-SY5Y cells was evaluated after treatment with Aβ_42_ ADDLs over time, with or without Ca^2+^ in the cell medium. The absence of extracellular Ca^2+^ determined levels of ROS similar to those observed in untreated cells up to 180 min of treatment with the oligomers, without any initial increase at early time points (Fig. 8c,d), indicating that the cells without any Ca^2+^ influx and dyshomeostasis did not undergo any oxidative stress, despite the treatment with toxic Aβ_42_ ADDLs in the absence of antioxidants (Fig. 8c,d). These results emphasise that while the suppression of the Ca^2+^ influx in the cells suppresses the oxidative stress for the entire length of time of the analysis, the cellular protection by a reducing agent does not suppress the initial oligomer-induced Ca^2+^ influx.

To confirm these results with different probes of intracellular Ca^2+^ and ROS, we repeated the experiments with ADDLs after 10 and 60 min of treatment, with or without Trolox and with or without Ca^2+^ in the cell medium, using the X-Rhod-1 AM and the CellRoxTM Deep Red Reagent to monitor Ca^2+^ and ROS levels, respectively. The results confirm that the presence of the antioxidant allowed the cells to react to and normalise the initial Ca^2+^ influx observed after 10 min of treatment, which reached the levels of untreated cells after 60 min of treatment (Fig. S3a,b), and that ROS levels remained constant and similar to those of untreated cells when the treatment was performed in a medium without Ca^2+^ (Fig. S3c,d).

**Fig. 9:**
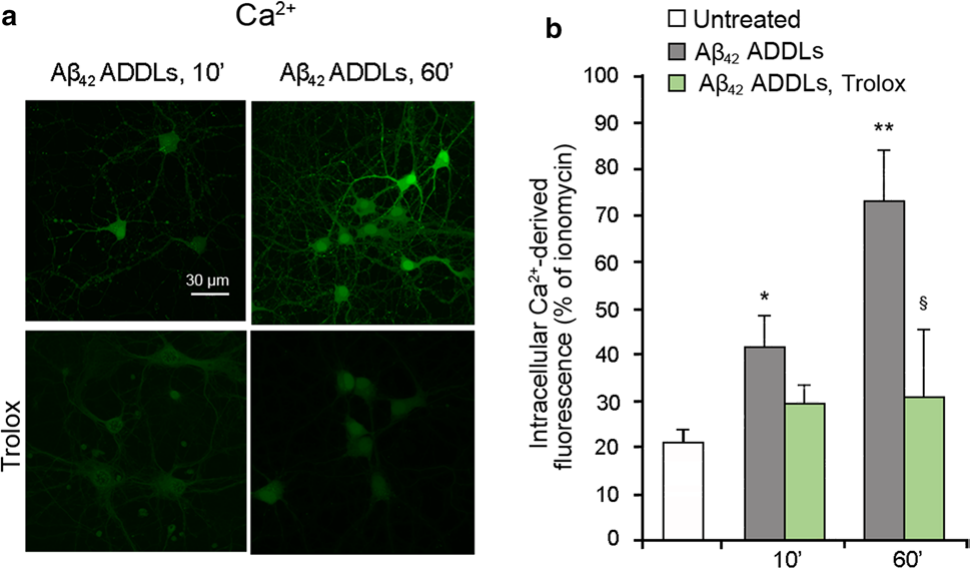
Intracellular Ca^2+^ influx and ROS production induced by Aβ_42_ ADDLs are connected in primary rat cortical neurons. **a** Representative confocal scanning microscopy images of intracellular free Ca^2+^ levels in primary rat cortical neurons with no treatment (first row), and pre-treatment with 30 µM Trolox (second row), and analysed after 10 and 60 min of treatment with 1 µM (monomer equivalents) Aβ_42_ ADDLs oligomers. **b** Semi-quantitative analysis of intracellular free Ca^2+^-derived fluorescence. Three different experiments were carried out, with 10–22 cells each, for each condition. Data are represented as mean ± SEM (*n* = 3). The single (*) and double (**) asterisks refer to *p* values < 0.05 and < 0.01, respectively, relative to untreated cells. The single (§) symbol refers to *p* values < 0.05 relative to Aβ_42_ ADDLs oligomers without pre-treatment with Trolox at corresponding time points

The effect of Trolox was also tested on primary rat cortical neurons. The cells were treated with Aβ_42_ ADDLs for 10 or 60 min, with or without the 1 h pre-treatment with Trolox. Similarly to SH-SY5Y cells, the presence of Trolox did not prevent a slight increase of the intracellular Ca^2+^ concentration, but caused lower levels of Ca^2+^ after both 10 and 60 min of treatment with Aβ_42_ ADDLs relative to cells pre-treated with Trolox (Fig. 9). This suggests that also in this cellular system the oxidative stress reduction allows the cells to counteract the initial Ca^2+^ influx across the membrane and restore the normal levels of Ca^2+^.

## Discussion

Dysregulation of Ca^2+^ signalling and excessive production of intracellular ROS are common early features of neurodegenerative disorders, in particular AD [13, 15, 47, 48]. Several studies have shown that the passage from the extracellular space into the cytosol of small molecules and ions, such as Ca^2+^ ions, is mediated by the interaction of Aβ oligomers, characteristic of AD, with the lipid bilayer [7, 11, 14, 17, 18]. Our results confirm these observations. They also confirm that this early modification is associated with the increase of cytosolic ROS levels. Interestingly, the increase of ROS production appears to occur more rapidly following the treatment with Aβ_42_ ADDLs than HypF-N OAs, which can be attributed to the known oxidative potential of Aβ_42_ ADDLs through Ca^2+^-independent mechanisms [44–46].

The maintenance of the Ca^2+^ gradient across the cell membrane, where the Ca^2+^ concentration is 50–100 nM inside the cell and 1.1 mM outside, represents a great energetic expense, because the plasma membrane Ca^2+^-ATPase (PMCA) and the sarco/endoplasmic reticulum Ca^2+^-ATPase (SERCA) need ATP to pump out the ions from the cytosol and restore homeostasis [6, 28, 36, 37]. Therefore, the increased need for ATP caused by the oligomer-induced Ca^2+^ dyshomeostasis activates the Krebs cycle, electron transport chain and oxidative phosphorylation in mitochondria, which determines the mitochondrial generation of ROS through the increased O_2_ reduction [28, 37]. ROS can also be produced by extramitochondrial enzymes, such as NADPH oxidase, xanthine oxidase, cytochrome P450, myeloperoxidase, cyclooxygenase, lipoxygenase and uncoupled nitric oxide synthase, all of which are modulated by Ca^2+^ [37]. This explains the association between Ca^2+^ dysregulation and increased ROS production.

The kinetic results presented here show that the delay in ROS production, which is evident as a lag phase and slower overall process in both time courses of ROS production following Aβ_42_ ADDLs and HypF-N OAs addition, is suggestive, albeit not a demonstration per se, that the ROS increase follows, and is caused by, that in Ca^2+^. To address further the cause-and-effect relationship between these two events, we took into consideration the data obtained with inhibitors of the Ca^2+^ influx and the known relationship between the two processes. Indeed, the extracellular-to-cytosol influx of Ca^2+^ induced by misfolded protein oligomers arises, at least in its early stages, from the passage of the ions through the AMPA and NMDA receptors [9, 15, 18, 19], which are mechanically activated following the modification of the phospholipid bilayer induced by the oligomers [14]. The pharmacological inhibition of the two glutamatergic receptors, with CNQX and memantine, respectively, delayed transiently the Ca^2+^ influx induced by these oligomers, with no significant increase within the first minutes of treatment. The delay mediated by CNQX and memantine, however, did not only involve the Ca^2+^ influx, but also ROS production. We also observed a delay in ROS levels increase following the pre-treatment with the inhibitors and this delay was even larger than that observed for Ca^2+^ levels. These results suggest that the inhibition of AMPA and NMDA receptors, with the consequent reduction of the early Ca^2+^ influx, allowed the cells to postpone ROS production. At later time points, intracellular Ca^2+^ levels increase despite the persistent inactivation of the two receptors, reaching the same levels observed in the absence of any inhibition, because it is caused by the direct passage of the ions through the cell membrane after the interaction of the oligomers with the lipid bilayer and a consequent destabilization and perforation [17, 18]. In addition, the Ca^2+^ pumps are inhibited by ROS, contributing to increase Ca^2+^ levels at later time points (see below). In the same way, ROS production increases, while continuing to maintain this slight delay because of the inactivation of the AMPA/NMDA receptors. The kinetic data, in particular, indicate that the use of either CNQX or memantine, or both, results in a lag time of the increase in Ca^2+^ levels, followed by the extension of the lag phase in ROS production. Other Ca^2+^ channels are probably involved in the oligomer-mediated Ca^2+^ influx, such as TRPM2 [23], VDCCs [24] and TRPA1 [25], but these have not been found previously to be involved significantly in our system [14]. The delay caused by CNQX and Memantine on Ca^2+^ and ROS kinetics was lower on cells treated with Aβ_42_ ADDLs, compared to cells treated with HypF-N OAs, probably because the damaging action of the first type of oligomer on the membrane is stronger, and, after its interaction with the lipid belayer and the consequent destabilization and perforation, the direct passage of the ions through the cell membrane is more pronounced compared to that of HypF-N OAs.

In AD brains, high levels of intracellular ROS were found to react with several macromolecules, such as proteins, polysaccharides, nucleic acids and lipids, causing their oxidation and leading to the production of reactive ketone/aldehyde moieties and other carbonyl derivatives [48]. An important deleterious effect of ROS in the brain is lipid peroxidation, which directly damages biological membranes [44, 49]. Moreover, high levels of ROS cause the oxidation of multiple methionine residues of the Ca^2+^ signalling protein calmodulin (CaM), with its consequent inability to activate its target proteins, including the PMCA, which is important for the maintenance of Ca^2+^ homeostasis [36, 50, 51]. High levels of ROS also result in tyrosine and cysteine oxidation of the SERCA [52–54]. Indeed, upon treatment with the antioxidant agent Trolox in our experiments, which completely inhibits the increase of ROS levels and prevents its damaging effects, it is likely that the cells are able to restore Ca^2+^ homeostasis effectively, as a result of the lack of ROS-mediated oxidation of the PMCA and SERCA, among other cellular factors.

The selective oxidation and inactivation of the Ca^2+^ regulatory proteins mediated by ROS may represent an adaptive response to the oxidative stress, because it down-regulates ATP production through the mitochondrial electron transport chain and the inevitable generation of ROS associated with it [28].

Further evidence of the importance of Ca^2+^ influx in ROS production occurs in the treatment with the oligomers in a medium without Ca^2+^ (to inhibit Ca^2+^ influx) and with an antioxidant agent (to inhibit ROS production). The absence of Ca^2+^ in the extracellular medium fully inhibits the increase of the intracellular Ca^2+^ levels that normally flow from the extracellular space, but at the same time fully inhibits ROS production, confirming that ROS result from the need to restore Ca^2+^ homeostasis. By contrast, treatment with the antioxidant agent Trolox leads the restoration of Ca^2+^ homeostasis, but only at prolonged time points. This latter analysis showed that Ca^2+^ ions enter the cells in the first minutes, because the antioxidant agent inhibits only ROS production and is not able to inhibit the oligomer-mediated activation of AMPA and NMDA receptors that occurs within the first minutes of interaction of the oligomers with the cell membrane. This rapid increase of intracellular Ca^2+^ is followed by a decrease, suggesting that the cells are able to pump out Ca^2+^ and restore homeostasis as they benefit from an effective antioxidant capacity induced by Trolox and absence of any direct ROS-induced inhibition of the PMCA, SERCA, other pumps and possibly other cellular factors involved in these processes.

## Conclusions

Vicious cycles, or positive feedback loops, exist between Ca^2+^ signalling and ROS production [28, 37], and even between Aβ production and Ca^2+^ signalling [55] and between Aβ production and ROS production [55], where the various events sustain each other. However, a precise cause-and-effect relationship between increased levels of intracellular Ca^2+^ and cytosolic ROS production at the very early stages of the overall dysregulation induced by misfolded protein oligomers emerges from our results by three distinct lines of evidence, namely: (i) a lag time observed in the time course of oligomer-induced ROS production (but not in Ca^2+^ increase), (ii) an ability of AMPA/NMDA receptor inhibitors to retard ROS production even more effectively than Ca^2+^ influx and (iii) an inability of antioxidant agents to inhibit the early Ca^2+^ influx, while fully maintaining the redox status of the cells, whereas a Ca^2+^ deprived medium inhibits fully and effectively both Ca^2+^ influx and ROS production. Hence, the oligomers cause the entry of Ca^2+^ ions in the cells, determining the formation of ROS due to the increased demand of ROS-generating ATP production by mitochondria; ROS in turn prevent the cells from pumping back Ca^2+^ ions into the extracellular space and from restoring the normal Ca^2+^ homeostasis, indicating a positive feedback on Ca^2+^ dyshomeostasis on the longer time scale.

### Supplementary Information

Below is the link to the electronic supplementary material.

Supplementary file1 (DOCX 1182 KB)

## Data Availability

Data will be made available on reasonable request.
