# Supplementary material for: Misfolded protein oligomers induce an increase of intracellular Ca2+ causing an escalation of reactive oxidative species
Source: Cell Mol Life Sci. 2022 Aug 27;79(9):500. doi: 10.1007/s00018-022-04513-w (PMC9420098; doi:10.1007/s00018-022-04513-w)
Supplement: Supplementary file 1 — Supplementary file1 (DOCX 1182 KB) [file 18_2022_4513_MOESM1_ESM.docx]

**An intracellular Ca^2+^ increase induced by misfolded protein oligomers enhances the production of reactive oxidative species**

Giulia Fani^1,2^, Chiara Ester La Torre^1^, Roberta Cascella^1^, Cristina Cecchi^1^,

Michele Vendruscolo^2*^, Fabrizio Chiti^1*^

*^1^Department of Experimental and Clinical Biomedical Sciences,*

*Section of Biochemistry, University of Florence, 50134 Florence, Italy.*

*^2^Centre for Misfolding disease, Department of Chemistry,*

*University of Cambridge, Cambridge CB2 1EW, UK.*

** To whom correspondence should be addressed:*

*Fabrizio Chiti: Department of Experimental and Clinical Biomedical Sciences, University of Florence, Florence, Italy, Viale Morgagni 50, 50134, Florence, Italy. E-mail:* [*fabrizio.chiti@unifi.it*](mailto:fabrizio.chiti@unifi.it)

*Michele Vendruscolo: Centre for Misfolding disease, Department of Chemistry, University of Cambridge, Cambridge CB2 1EW, UK. E-mail:* [*mv245@cam.ac.uk*](mailto:mv245@cam.ac.uk)

**Supplementary Material**


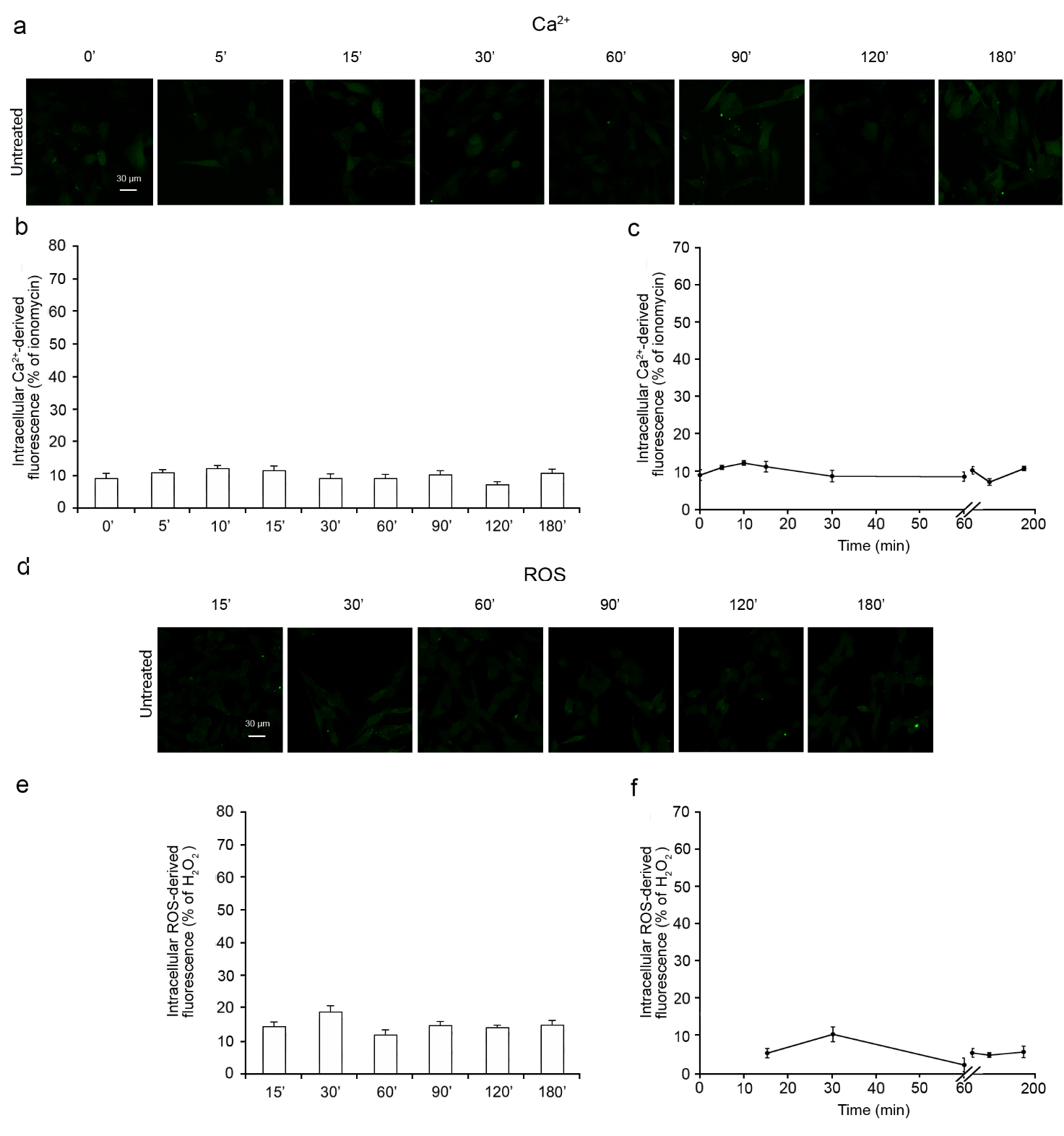


**Figure S1. Basal intracellular Ca^2+^ and ROS levels. (a)** Representative confocal scanning microscopy images of free Ca^2+^ levels in SH-SY5Y cells following the treatment with cellular medium for 5, 10, 15, 30, 60, 90, 120 and 180 min. **(b)** Semi-quantitative analysis of intracellular free Ca^2+^-derived fluorescence. **(c)** Kinetic plot showing the fluorescence *versus* time as reported in panel B. **(d)** Representative confocal scanning microscopy images of intracellular ROS levels in SH-SY5Y cells following the treatment with cellular medium for 5, 10, 15, 30, 60, 90, 120 and 180 min. **(e)** Semi-quantitative analysis of intracellular ROS-derived fluorescence. **(f)** Kinetic plot showing the fluorescence *versus* time as reported in panel E. Three different experiments were carried out, with 10-22 cells each, for each condition. Data are represented as mean ± SEM (n=3)


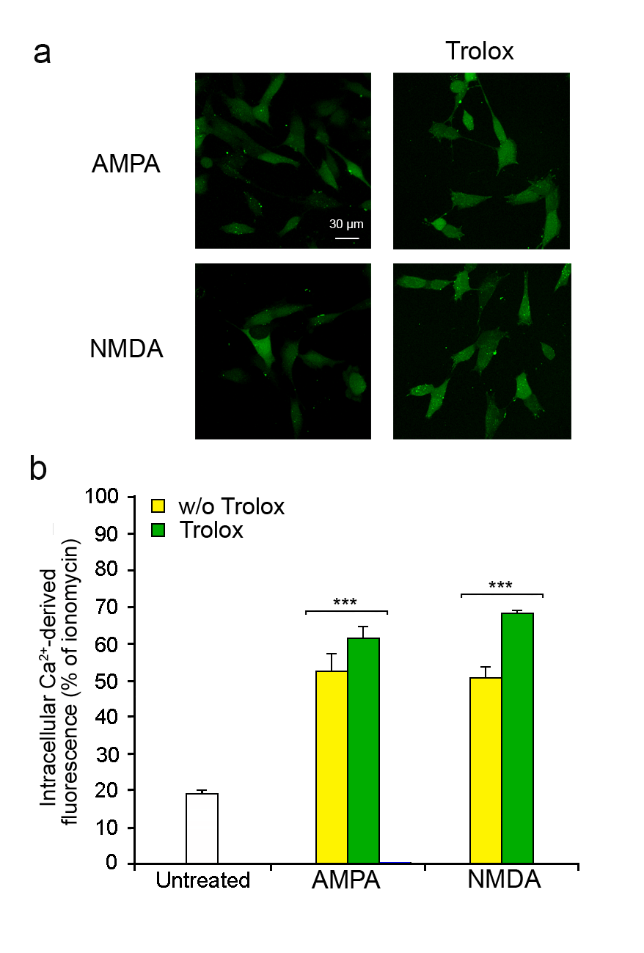


**Figure S2. Lack of dependence of the intracellular Ca^2+^ influx induced by AMPA and NMDA upon Trolox pre-treatment. (a)** Representative confocal scanning microscopy images of intracellular free Ca^2+^ levels in SH-SY5Y cells following treatment with 50 µM AMPA (first row) and 1 mM NMDA (second row), in the presence (second column) and absence (first column) of a 1 h pre-treatment with 30 µM Trolox. **(b)** Semi-quantitative analysis of intracellular free Ca^2+^-derived fluorescence. Three different experiments were carried out, with 10-22 cells each, for each condition. Data are represented as mean ± SEM (n=3). The triple (***) asterisks refer to p values <0.001 relative to untreated cells


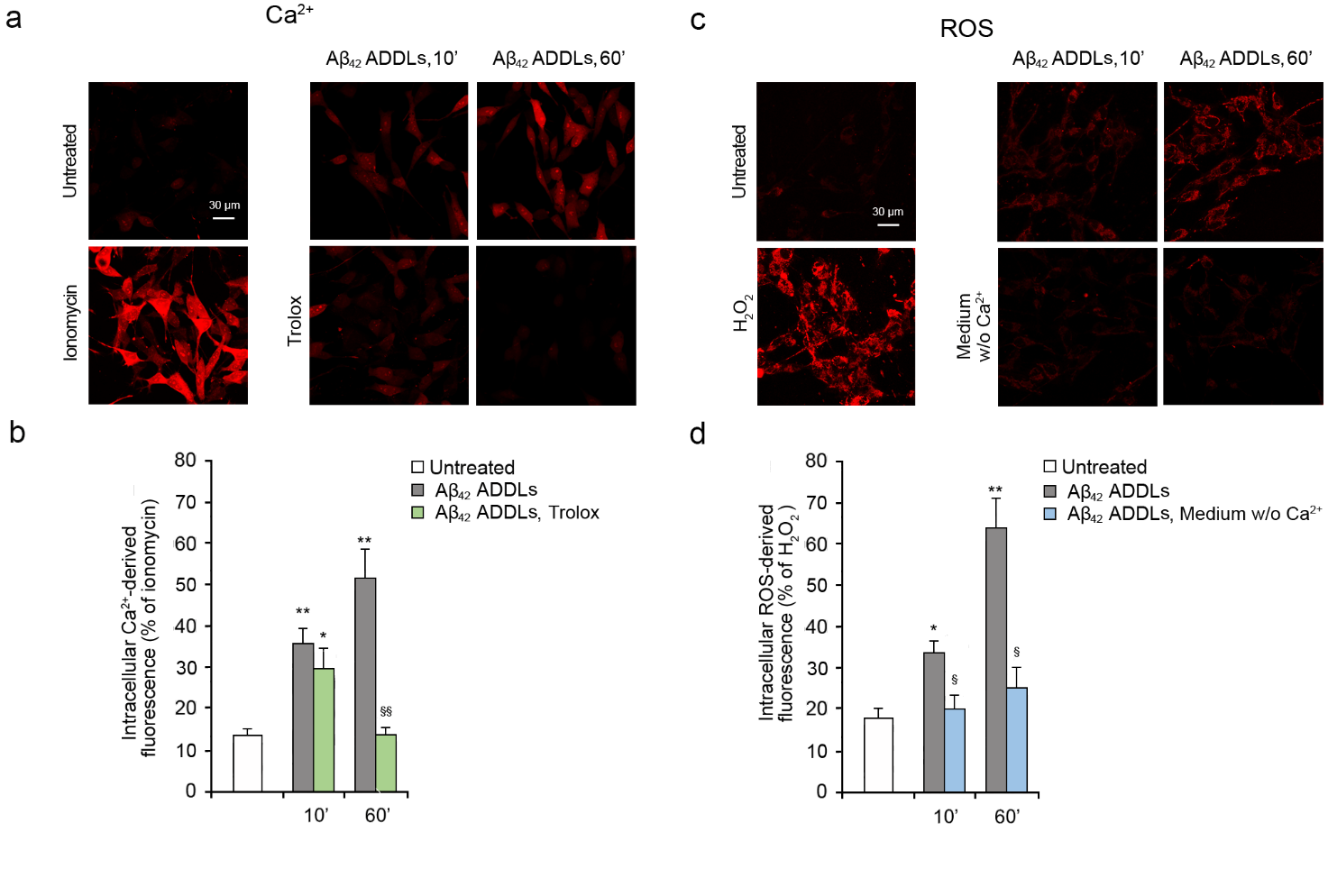


**Figure S3.** **Intracellular Ca^2+^ influx and ROS production induced by Aβ_42_ ADDLs are connected in SH-SY5Y cells when monitored with different probes.** **(a)** Representative confocal scanning microscopy images of intracellular free Ca^2+^ levels in SH-SY5Y cells following no treatment, treatment with 1 µM ionomycin for 1 h without Aβ_42_ ADDLs, pre-treatment without (first row) or with (second row) 30 µM Trolox and analysed after 10 and 60 min of treatment with 1 µM (monomer equivalents) Aβ_42_ ADDLs oligomers. **(b)** Semi-quantitative analysis of intracellular Ca^2+^-derived fluorescence. **(c)** Representative confocal scanning microscopy images of intracellular ROS levels in SH-SY5Y cells following no treatment, treatment with 250 µM H_2_O_2_ for 1 h without Aβ_42_ ADDLs, and treatment in a medium with Ca^2+^ (first row) or without Ca^2+^ (second row), and analysed after 10 and 60 min of treatment with 1 µM (monomer equivalents) Aβ_42_ ADDLs oligomers. **(d)** Semi-quantitative analysis of intracellular ROS-derived fluorescence. Three different experiments were carried out, with 10-22 cells each, for each condition. Data are represented as mean ± SEM (n=3). The single (*) and double (**) asterisks refer to p values <0.05 and <0.01, respectively, relative to untreated cells. The single (§) and double (§§) symbols refer to p values <0.05 and <0.01, respectively, relative to Aβ_42_ ADDLs oligomers without treatment with Trolox or Ca^2+^-deprived medium at corresponding time points
